# Recurrent fever: diagnostic procedure

**DOI:** 10.1186/1824-7288-40-S1-A25

**Published:** 2014-08-11

**Authors:** Elisabetta Cortis

**Affiliations:** 1 Department of Pediatrics - USL 2 - 06034 Orvieto (TR), Italy

## 

Fever is defined periodic/recurrent in presence of three or more episodes of fever of unknown origin that occur in a period of six months and whose onset is at least one week apart from each other. The diagnosis, often complex, should exclude an infectious cause and an onco-haematological disease.

History and physical examination are essential to make a diagnostic hypothesis of a periodic fever. A recurrence of febrile episodes throughout the year including the summer period, intervals of complete wellbeing, fever associated with symptoms and similar clinical signs, length of episodes and similar intervals, spontaneous resolution of symptoms, would suggest a periodic fever. The inflammatory markers (ESR, CPR, SAA) and the number of WBC increase in the period of fever and tend to normalize with fever resolution [1]. FPAFA syndrome, the most common, is characterized by episodes of fever, that occur under 5 years of age, are accompanied by cervical lymphadenitis, pharyngitis, mouth aphtas; in the febrile phase, the child keeps good general conditions. Steroid therapy has proven effective in the resolution of fever, even if it has been demonstrated that the regular intake of cortisone at any fever onset may lead to a shortening of the intervals. In the differential diagnosis, due consideration should be given to cyclic neutropenia that is a genetic disease (mutation of ELA-2 gene) characterized by fever every 3-4 weeks (it appears with a decrease of neutrophils), pharyngotonsillitis and aphtas larger than those of PFAPA, and often severe bacterial infections. In such cases, a complete blood count performed every week for at least four weeks may be useful to make the proper diagnosis PFAPA does not require further diagnostic investigations and prognosis is benign with spontaneous resolution within 10 years of age. For the monogenic periodic fevers that are FMF, TRAPS and MKD, fever is accompanied by a systemic involvement such as rash, arthritis, serositis (table 1). In the febrile period the child has a general malaise. Ethnicity, age of fever onset, duration of fever vary between one form and another. In order to make easier the identification of a periodic monogenic fever a diagnostic score has been developed that can predict the risk that a pediatric patient may have one of these forms. The score takes into consideration the following variables: age of fever onset, abdominal pain, mouth aphtas, diarrhea, positive family history [2].

**Table 1:** Main clinical manifestations during fever episodes in patients with periodic fever syndrome and genes responsible of hereditary periodic syndrome.

	FMF	TRAPS	MKD/ HyperIgD	PFAPA
Abdominal pain	+++	+++	++	-

Diarrhea	+/-	-	+++	-

Arthritis	+++	-	+++	

Lymphadenopathy	+/-	++	+++	+++

Rash	+/-	++	+++	-

Stomatitis	-	-	-	+++

Pharyngitis	-	-	-	+++

Splenomegaly	++	-	+++	+/-

Conjunctivitis	-	+++	-	-

Duration of fever	1-4 Days	Variable >1 week	2-5 Days	2-8 Days

Gene/chromosome	MEFV16p13.3	TNFRSF1A12p13	MVK 12q24	unknown

Protein	Pyrin	TNFR1	MVK	unknown

Inheritance	AR	AD	AR	none
